# Prevalence of ZnT8 Antibody in Turkish Children and Adolescents with New Onset Type 1 Diabetes

**DOI:** 10.4274/jcrpe.5020

**Published:** 2018-05-18

**Authors:** Selin Elmaoğulları, Seyit Ahmet Uçaktürk, Şehri Elbeg, Esra Döğer, Meltem Tayfun, Fatih Gürbüz, Aysun Bideci

**Affiliations:** 1 University of Health Sciences, Ankara Children’s Hematology and Oncology Training and Research Hospital, Clinic of Pediatric Endocrinology, Ankara, Turkey; 2 Gazi University Faculty of Medicine, Department of Biochemistry, Ankara, Turkey; 3 Gazi University Faculty of Medicine, Department of Pediatric Endocrinology, Ankara Turkey

**Keywords:** ZnT8 antibody, children, adolescent, type 1 diabetes

## Abstract

**Objective:**

Zinc transporter 8 protein (ZnT8A) is a transmembrane protein which functions to transfer zinc to insulin vesicles. Antibodies formed against ZnT8A (ZnT8A) are regarded as an independent autoimmunity demonstrator in type 1 diabetes (T1D). The aim of this study was to investigate the prevalence of ZnT8A in Turkish children with new onset T1D.

**Method:**

Eighty four patients between 1-18 years of age diagnosed with T1D between February 2015-March 2016 and the control group consisting of 50 healthy children without any autoimmune diseases were included in the study. Serum samples for ZnT8A testing were taken from the patient group within a week of diagnosis. A ZnT8A enzyme-linked immunosorbent assay was used in the analyses.

**Results:**

ZnT8A positivity was detected in 58% of the patients with new onset T1D and 8% of the control group. ZnT8A were demonstrated in 5 of 11 patients with negative results for classical diabetes antibodies [insulinoma antigen-2 antibody (IA-2A), glutamic acid decarboxylase (GAD) or insulin autoantibodies]. No association was found between ZnT8A positivity and age, gender, presence or degree of ketoacidosis at presentation, hemoglobin A1c, insulin or C-peptide concentration, or the presence of either thyroid or celiac antibodies.

**Conclusion:**

ZnT8A prevalence in children with T1D in Turkey was compatible with the literature. The ratio of patients who are clinically considered to have T1D but have negative routine diabetes auto-antibodies were observed to decrease nearly by 50% when ZnT8 antibodies were added to the panel. ZnT8 measurement should be more widespread for clarifying the etiology in T1D.

## What is already known on this topic?

The presence of zinc transporter 8 protein antibodies in diabetic children with type 1 diabetes changes according to countries. The prevalence is reported to range between 24% and 80%.

## What this study adds?

Zinc transporter 8 protein (ZnT8A) antibody positivity was 58.2% in Turkish children with type 1 diabetes. ZnT8A antibody was present in 46.6 % of cases with which were negative for classic type 1 diabetes associated autoantibodies.

## Introduction

Type 1 diabetes (T1D) is a chronic disease characterized by immune-mediated, selective destruction of pancreatic beta cells (1). Decrease in the induction effect of infections on negative immuno-regulatory genes, due to genetic predisposition and also due to a more hygienic way of life, which has become more significant in the last few decades, probably plays a role in the initiation of immune mediated degradation (2). The presence of DRB1*04-DQB1*0302 and DRB1*03 among human leukocyte antigen (HLA) class II haplotypes in at least 90% of the individuals with the disease, detection of autoreactive anti-islet antigen specific T cells in the circulation of new onset or prediabetic individuals, demonstration of lymphocyte infiltration in the islet cells during the development of insulitis and increased predisposition to Addison’s disease and celiac disease support the role of autoimmunity in the progression to T1D (2,3,4,5,6).

Although the initial steps stimulating the autoreactive cascade are unknown, it is suggested that autoreactive and cytotoxic T cells, activated by presentation of pancreas antigens to T cells by antigen presenting immune cells, cause beta cell destruction (7,8). Islet cell antigen (ICA), glutamic acid decarboxylase (GAD) 65, insulin and insulinoma antigen-2 (IA-2) are the main well-defined pancreatic antigens. Antibodies formed against some or all of these antigens are positive in more than 80% of new onset T1D patients (9). However, the increasing number of studies on new pancreas antigens, such as zinc transporter 8 (ZnT8), pancreatic duodenal homeobox factor-1, chromogranin A and islet amyloid polypeptide, may possibly lead to development of new treatment options and clarify etiology in idiopathic T1D patients (9,10,11,12). 

Zinc is essential for the structural stabilization of insulin. Pancreas is one of the tissues with the highest zinc concentration. Zinc transportation to insulin vesicles is mediated by ZnT8, a transmembranic protein (13). It is encoded by the *SLC30A8* gene located in 8q24.11 (14). Antibodies formed against ZnT8 (ZnT8A) are regarded as an independent autoimmunity demonstrator in T1D diagnosis (9). When used in combination with IA-2 antibody (IA-2A), their predictivity for T1D and cost-effectivity compared to other antibody combinations is higher in “at risk” individuals, regardless of their age (15). Although their prevalence in children with new onset T1D changes by country and study, it is reported to be 24-80% and it is suggested that the presence of ZnT8A be investigated in all diabetes patients regardless of their ethnicity (16,17,18). ZnT8A presence has been shown in nearly 25% of patients accepted as idiopathic T1D who were negative for the classic autoantibodies (9,15). This study aimed to investigate ZnT8A prevalence in Turkish children with new onset T1D and the relation of ZnT8A to other antibodies.

## Methods

A total of 84 patients, between 1-18 years of age, diagnosed with T1D in Ankara Pediatric Hematology Oncology Training and Research Hospital (n=76) and in Gazi University Faculty of Medicine (n=8) between February 2015 and March 2016 composed the subject group. Fifty healthy children with no autoimmune diseases were included in the study as controls (19).

Presence and degree of ketosis or ketoacidosis were recorded at the time of referral (pH 7.3-7.2 mild; 7.2-7.1 moderate <7.1 severe ketoacidosis). C-peptide concentration was determined in serum samples taken during diagnosis, using the *chemiluminescence* immunoassay method. Patients with a C-peptide level above 1 ng/mL were excluded from the study. Hemoglobin A1c (HbA1c) was determined by immune turbidimetry using a modular P800 analyser (Roche Diagnostics, Basel, Switzerland). Cut-off for positivity for the following antibodies were: GAD antibody (GADA) concentration above 1 IU/mL; IA-2A concentration above 1 U/mL (both tested using radioimmunoassay method); IA concentration above 0.4 U/mL and anti-tissue transglutaminase IgA (tTG IgA) above 18 U/mL [both tested using the micro Enzyme-Linked ImmunoSorbent Assay (ELISA) method]. Thyroid function tests were performed following a ketotic or ketoacidotic period, after establishing euglycemia in the patients. Thyroid stimulating hormone (TSH) and free T4 (fT4) levels were determined using the two-region, two-stage enzymatic immunoassay method. According to the reference values of the TSH and fT4 kits, TSH lower and upper limit values were accepted as 0.34-5.6 mIU/mL and fT4 lower and upper limits as 0.6-1.2 ng/dL. Anti-thyroid peroxidase (anti-TPO) and anti-thyroglobulin (anti-TG) antibodies were measured using the Beckman Coulter DX1800 chemiluminescence immunoassay method.

The ELISA method was used to determine ZnT8A concentration in serum samples which were taken within a week after diagnosis and stored at -80 °C. Medizym anti ZnT8 ELISA kit, which can detect antibodies to arginine (R-325), tryptophan (W-325) and other non-specific variants, was used to test for the presence of these antibodies. Concentrations above 15 U/mL were accepted as positive.

Ethic board consent for the study was granted by the ethic board of Ankara Pediatrics Hematology Oncology Training and Research Hospital (consent number: 2015-002). All parents were informed about the purpose of the study, and a signed consent for study participation was obtained.

### Statistical Analysis

Statistical analysis of the data was carried out using “The Statistical Package for the Social Sciences 17.0” (SPSS, Inc. Chicago IL, USA, Microsoft) programme. Results were expressed as mean ± standard deviation for parametric data and median + range for nonparametric data. The Student t-test was used for the comparison of the medians for numeric variables and the chi-square test for comparing the medians for non-numeric variables. Mann-Whitney U test was preferred for the evaluation of numeric parameters without a normal distribution. Significance level was accepted as p<0.05.

## Results

The mean age of the 84 (49 female, 35 male) cases with T1D was 9.8±4.0 years and 52.4% were prepubertal children. In the control group (25 females, 25 males) the mean age was 9.1±4.0 years. Twenty-two cases (26.2%) had been referred with hyperglycemia, 23 (27.4%) with ketosis, 39 (46.4%) with ketoacidosis (12 mild, 8 moderate and 19 severe degrees of ketoacidosis). Mean HbA1c was 11.7±2.3% and C-peptide level 0.41±0.29 ng/mL. Accompanying hypothyroidism was not observed in any of the patients (TSH: 2.53±1.2 mIU/L; fT4: 0.97±0.22 ng/dL).

**Figure 1:**
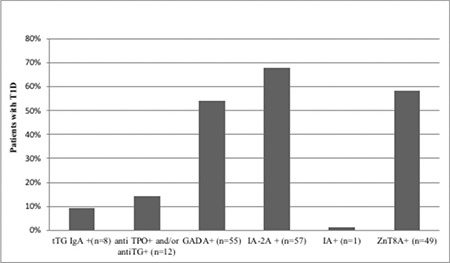
Frequency of celiac, thyroid and diabetic autoantibodies in 84 patients with new onset type 1 diabetes T1D: type 1 diabetes, ZnT8A+: zinc transporter 8 protein positive, tTG: anti-tissue transglutaminase, IgA: immunoglobulin A, TPO: thyroid peroxidase, TG: thyroglobulin, GADA: glutamic acid decarboxylase antibodies, IA: insulinoma antigen, IA-2A: insulinoma antigen-2 antibody

ZnT8A positivity was detected in 49 (58.2%) cases in the T1D patient group and in four (8%) individuals in the control group. ZnT8A was present in five out of 11 (13%) of cases all of which were negative for GADA, IA-2A and IA. Prevalence figures for tTG IgA, anti-TPO and/or anti-TG and diabetes autoantibodies in T1D patients are depicted in Figure 1. When ZnT8A positive (ZnT8A+) and ZnT8A negative (ZnT8A-) T1D cases were compared, no difference was detected in age, gender, presence and degree of ketoacidosis during referral, HbA1c concentration, insulin or C-peptide concentrations. When they were compared for prevalence of celiac, thyroid and other diabetes autoantibodies, it was observed that only the IA-2A positivity rate was significantly higher in ZnT8A+ cases with T1D (p=0.024) (Table 1). It was also observed that ZnT8A titers in ZnT8A+ cases in T1D group were significantly higher (median 271.37 U/mL, range 23.28-501.00 U/mL) compared to the titres of the ZnT8A+ cases in the control group (median 26.96 U/mL, range 15.1-93.9 U/mL). While ZnT8A titer was not found to be related to age and body mass index (BMI), a week positive correlation was detected with C-peptide level (p=0.034, r=0.31). None of the four ZnT8A+ patients among control group were diagnosed T1D within two-year follow. Further follow-up is planned in these patients.

## Discussion

This study showed that the prevalence of ZnT8A is 58.6% in Turkish children with new onset T1D. This result is in accordance with most of the studies done in other countries. ZnT8A positivity was reported to be between 60-80% in Caucasians (1-18 years old) (9), 72 % in Czechs (1-19 years old) (20) and 65% in Argentinians (10-32 years old) (21), with new onset T1D. ZnT8A positivity was reported in 24% of Chinese new onset T1D patients (1-70 years old) and differences in HLA genotypes or other inter-ethnic genetic markers were thought to be a possible cause for this low rate (17). However, in another Asian population, Japanese acute onset T1D patients (19.1±14.5 years old) had 58% ZnT8A positivity, which is very similar to our findings in a Turkish population (22). A study from Brazil, which encompassed both a Caucasian and a non-Caucasian new onset T1D population (30.3±11.4 years old), found an overall ZnT8A positivity of 24% and it was stated that neither ZnT8A positivity nor concentration was associated with ethnicity (23). 

The ZnT8A positivity prevalence of healthy controls from different countries was reported as 1-2.7%, which is a markedly lower rate than in this study (8%) (9,17,20,24). This difference may be attributed to the larger cohorts of the other studies which better reflects the population. However, the possible effect of ethnicity cannot be excluded. In line with studies using the same analysis method and cut-off value, ZnT8A levels found in the control groups were lower than that found in the T1D patients (20,24). ZnT8A was shown to predict risk of progression to T1D in first degree relatives of T1D patients (15). Although these healthy controls had a negative T1D history in their families, they may have a higher risk for diabetes.

ZnT8A is directed to an epitope at the C terminal of the ZnT8 protein (residues 268-369). Gene polymorphism at the codon for the 325^th ^aminoacid lead to different variants of the ZnT8 protein and antibodies are found which are specific to each of these. These are R325-ZnT8RA, W325-ZnT8WA and rarely Glutamine (Q325)-ZnT8QA variants (9). It has been demonstrated that the distribution of antibody variants differs between populations (25). The methodology used in this study is capable of detecting autoantibodies against all three of these ZnT8 variants ZnT8A variant distribution in Turkish T1D children could not be determined. 

Prospective studies following up first-degree relatives of T1D patients or individuals with high risk HLA tissue types, starting from the first months of their lives until the development of T1D, demonstrated that ZnT8A developed many years prior to the development of T1D, in the 9^th^ month of life at the earliest and mostly close to three years of age (9,26). The youngest ZnT8A+ T1D case in our study was two years old. When present studies are considered, it may be predicted that the seroconversion starts around the age of one year. It was observed that ZnT8A+ prevalence and concentration during diagnosis did not change with age in our study. Andersson et al (27) reported that ZnT8A prevalence during diagnosis was age-independent in 686 children with T1D. In a study examining 227 children and adolescents with T1D, it was shown that ZnT8A prevalence was not related to age at diagnosis, while ZnT8A titers increased with age (20). In studies in which the subjects are older, it is observed that both ZnT8A prevalence and titer decreases with a higher age at diagnosis (16). 

In the present study, neither presence nor levels of ZnT8A were found to be related to BMI, a finding similar to some previous reports (16,17,18,19,20,21,22,23,24,25,26,27,28). Conversely, there are reports indicating that ZnT8A positivity is more frequent in the leaner T1D patients than in the more obese, but it was mentioned that larger cohorts were necessary to verify this negative association (17). 

Whether ZnT8A presence or levels predict residual beta cell function or not is still unclear, but there are reports indicating that the presence or levels of ZnT8 are unrelated to C-peptide levels (16,17). Andersson et al (27) found that both presence and levels of ZnT8RA and, to a lesser degree, ZnT8QA were associated with higher levels of stimulated C-peptide after diagnosis and during the follow-up of T1D. After excluding Znt8RA negative subjects and re-analyzing the relation between ZnT8A levels and stimulated C-peptide levels, that association failed to reach significance, a finding which may indicate that positivity rather than the level of Znt8RA has a protective role on beta-cell function (29). In contrast, the results of this present study showed that C-peptide levels were positively correlated with the concentration, but not with the presence of ZnT8A. Different cohort sizes, target ZnT8A epitope and type of C-peptide measurement, fasting or stimulated, may have caused these conflicting results.

### Study Limitations

The study is limited by a relatively small number of subjects and lack of ICA antibody measurement.

## Conclusion

ZnT8A is an independent marker of b-cell autoimmunity and its prevalence was found 58.6% in Turkish children with new onset T1D. Nearly half of the T1D patients negative for IA-2A, GADA and IAA were detected to be pozitive for ZnT8A which supports that ZnT8 measurement should be more widespread for clarifying the etiology in T1D.

## Figures and Tables

**Table 1:**
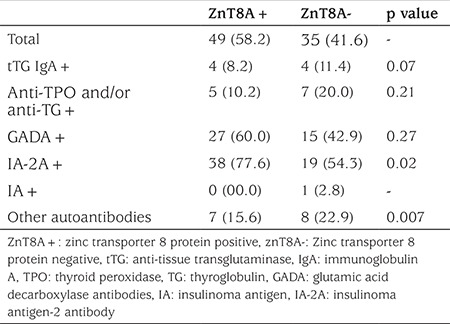
Prevalence of specific autoantibodies in zinc transporter 8 protein positive and zinc transporter 8 protein negative patients with new onset type 1 diabetes. Prevalences are given as n (%)
